# Prescription Department

**Published:** 1898-01

**Authors:** 


### PAROTITIS.

In a case of unilateral parotitis. with carious teeth, Dr. Eshner prescribed:

℞. Belladonna ointment............
Mercurial ointment..equal parts.

Mix. Apply topically morning and night.

And:

℞ Cinchonidin salicylate, 1 drachm.
Divide in twenty tablets.
Dose. One every three hours. —*Philadelphia Polyclinic.*

### ADMINISTRATION OF CREOSOTE.

The *Journal des Practiciens* recommends the following formula for the administration of creosote, the prescription being put up in cachets:

℞. Creosote........................
Benzoin.......of each 15 grains.
Powdered charcoal..$1\frac{1}{2}$ drachms.

Triturate the creosote and the benzoin for a moment together and add by degrees the charcoal. This mass is then to be divided into five or ten cachets, each one of which will contain a proper dose. It is claimed that this prescription is very well borne by the stomach.

